# Effects of a Dominant Species on the Functional Diversity of Coexisting Species in Temperate Deciduous Understorey

**DOI:** 10.3390/plants10112252

**Published:** 2021-10-21

**Authors:** Krishan Kaushik, Alessandro Bricca, Michele Mugnai, Daniele Viciani, Kinga Rudolf, Katalin Somfalvi-Tóth, Tamás Morschhauser

**Affiliations:** 1 Institute of Biology, University of Pécs, Ifjúság str. 6, 7624 Pécs, Hungary; morsi@gamma.ttk.pte.hu; 2 School of Biosciences and Veterinary Medicine, University of Camerino, Via Pontoni 5, 62032 Camerino, Italy; alessandro.bricca@unicam.it; 3 Department of Biology, University of Florence, Via G. La Pira 4, 50121 Firenze, Italy; michele.mugnai@unifi.it (M.M.); daniele.viciani@unifi.it (D.V.); 4 Institute of Plant Production Science, Campus of Szent István, University of MATE, 7400 Kaposvár, Hungary; rukinga@freemail.hu; 5 Department of Water Management and Climate Adaption, Institute of Environmental Science, Hungarian University of Agriculture and Life Sciences, 40. S. Guba str, 7400 Kaposvár, Hungary; somfalvi-toth.katalin@uni-mate.hu

**Keywords:** *Allium ursinum*, assembly rules, biotic interactions, clonal traits, functional traits, niche differentiation

## Abstract

The herb layer plays a significant role in maintaining forest functions, and its community composition is determined by various abiotic factors and biotic interactions. This study attempted to investigate the interspecific plant–plant biotic interactions using a functional traits approach. Specifically, the effects of a dominant species coverage on the functional diversity of coexisting species in the temperate forest understory were studied. Species coverage and soil moisture data were collected using a 1 m^2^ quadrat couplet (2 × 1 m^2^) from six sites alongside a 20 m linear transect encompassing a cover gradient of *Allium ursinum* in southwest Hungary. Major plant functional dimensions i.e., aboveground, and clonal functional traits were considered. Linear and nonlinear mixed models to quantify the effects of biotic interaction on the functional diversity of every single trait and multiple traits were employed. Both aboveground traits and clonal traits of persistent clonal growth organs responded positively to the *A. ursinum* L., cover gradient. The coexistence of understory species in the presence of a monodominant species seems to be mainly influenced by aboveground traits as compared to the clonal traits suggesting, a role of niche differentiation. The consistent impact of *A. ursinum* coverage on coexisting species dynamics highlights a need for similar in-depth studies in various forest settings.

## 1. Introduction

The understorey of temperate forest ecosystems has a significant impact on forest regeneration and conservation of biodiversity [1,2]. Albeit representing less than 1% of forest biomass, it may account for up to 90% of plant diversity [3]. Co-occurrences of understorey species are usually related to the accessibility of key abiotic ecological resources such as light, nutrients, and moisture etc., although light limitation has been found to have the most significant role in determining the herb layer’s diversity [4,5]. Similarly, interspecific biotic interactions of a dominant species (with strong “competitive effects traits”; Navas and Violle [6]) are associated with maximum resource acquisitions that could potentially further influence the species composition in a given community [7,8]. The dominant species supersedes the weaker competitors in a community by reflecting highly competitive behaviour such as a rapid vegetative growth rate [6,9,10]. However, owing to certain ecophysiological effects (nutrient richness, or high soil moisture content etc.) the abundances of some species might exceed the natural fluctuations (in this case they are defined as an “expansive species”; [11,12,13]), which subsequently can lead to the loss of biodiversity [14,15]. It might serve as an early warning sign for urgently taking measures in the conservation of a given ecosystem [16,17,18,19]. 

The largely overlooked biotic interaction such as interspecific competition influences forest floor diversity and its composition [20,21,22]. Thus, understanding its potential role in the assembling of herb layer community species still represents a challenge to be met [23,24]. The understanding of biotic interactions in the context of plant traits has been significantly improved using plant traits in recent decades [25,26]. The most recent use of traits has been reported by Kunstler et al. [27], using them to decipher the competition effects on community assembly processes across all the major biomes on Earth. The interspecific competition has been documented as one of the most determining factors which affect plant growth, survival, reproduction and also coexistence in a community [28,29]. Traits shed light on the underlying mechanisms of how species are organized into communities [30,31,32,33]. By definition, a functional trait is any measurable morphophysiological or behavioural characteristic of a plant that may influence its overall fitness [34].

As per contemporary assumptions, the composition of a plant community could be a result of stochastic or deterministic niche-based mechanisms [35,36]. Seemingly, negative biotic interactions (i.e., competition) could produce two contrasting outcomes [37]. On one hand, species coexistence might be achieved by processes of niche partitioning because there is a limit in niche overlapping (“limiting similarity”, MacArthur and Levins [38]), leading to an assemblage of species being functionally dissimilar (trait divergence). Alternatively, the prevalence of hierarchical differences in the competitive abilities among species should favour strong competitors over less competitive species, thus filtering out all those species with less competitive traits through the process called “competitive exclusion” [6,10,39]. This would result in the coexistence of species with similar trait values (functional convergence). To differentiate between these two assembly processes, we adopted a null model based on a functional traits matrix [40,41,42].

We considered the functional traits explaining the largest plant functional dimension for aboveground traits; referred to as the leaf-height-seed (LHS) scheme of Westoby [43] improved by Díaz et al. [44] and also clonal traits as per Klimesová et al. [45,46]. We focused on these key informative traits on different ecological functions such as resource economics (leaf dry matter content; henceforth ‘LDMC’ and leaf area; henceforth ‘LA’), competitive ability (vegetative height; henceforth ‘H’), sexual reproduction (seed mass; henceforth ‘SM’) [43,44], the clonal traits related to space occupation (lateral spread; henceforth ‘LS’) and resource foraging (persistence of clonal growth organ; henceforth ‘PCGO’ and the number of clonal offspring; henceforth ‘NCO’) [47].

The case studies of Heinrichs et al. [48] and those of Dierschke [49,50] have highlighted how a widespread, dominant species, *Allium ursinum* L., has become “an expansive species” in recent years in Germany where it formed homogenised stands causing serious nature conservation problem. In its native mesic forests, the species regularly forms monotypic stands due to competitive interactions with neighbouring species assemblages [51,52,53,54,55]. However, these direct competitive interactions could be mediated by either abiotic effect or abiotic-biotic (anthropogenic) effect complexes, but only a few plant-plant biotic interactions e.g., allelopathic influences etc. have been studied so far [13,56].

Therefore, this study attempts to investigate this underexplored biotic interaction aspect via an expansive species in a Hungarian temperate forest understorey. Study sites encompassed semi-natural forests under quasi-homogeneous abiotic ecological situations such as single vegetation type etc. and field data (coverage and moisture) were collected after adopting a stringent site selection criterion and using a fine-scale double quadrat along a transect having a cover gradient of *A. ursinum*. Functional traits data capturing the major functional dimension of plants (retrieved from databases) were used while strictly focusing on biotic interactions i.e., the effects of increasing coverage of *A. ursinum* were studied on ecological strategies of coexisting species in terms of functional traits [57]. 

We were interested in knowing whether (H1) *A. ursinum* coverage has effects on the functional diversity of coexisting species; (H2) Among biotic interactions, *A. ursinum* has different effects on the proportions or abundances of species’ above ground or clonal plant properties, or both. (H3) *A. ursinum* has different effects if the floristic compositions are different between the types of sites.

## 2. Results

### 2.1. Effects of A. ursinum Coverage on the Functional Diversity of Coexistence Species

**Figure 1:**
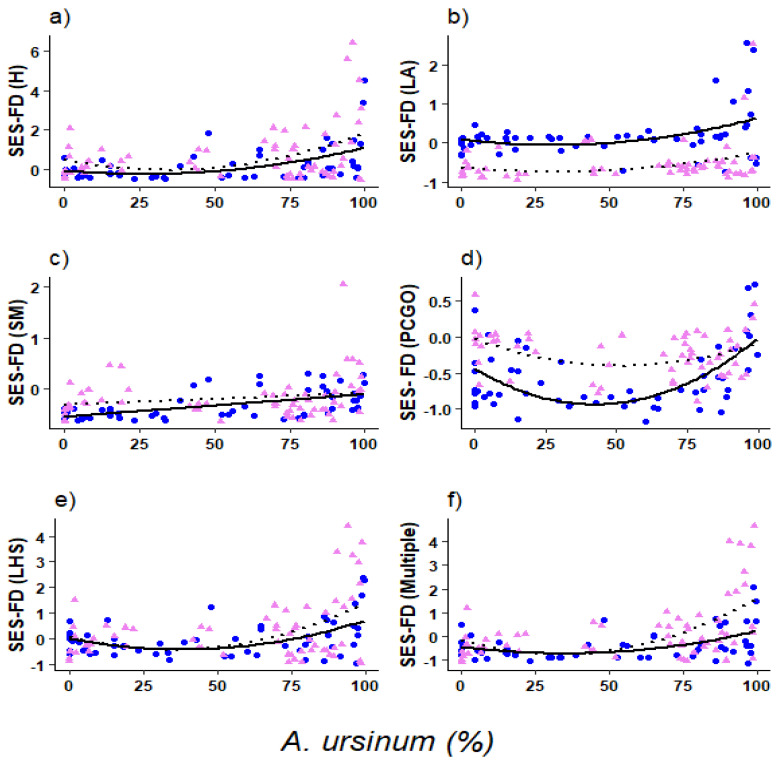
Results of the mixed model highlighting the effects of *A. ursinum* coverage on the standardized effect size of functional diversity (SES-FD) of: (**a**) plant height (H); (**b**) leaf area (LA); (**c**) seed mass (SM); (**d**) persistent clonal growth organ (PCGO); (**e**) leaf dry matter content, plant height and seed mass (LHS); (**f**) all studied traits combined i.e., aboveground and clonal traits together (Multiple). Pink triangles and black dashed lines represent the transect plot in sites where *M. uniflora* was present; blue circles and continuous black lines represent the transect plot where *C. pilosa* was present.

As a response to *A. ursinum* cover gradient, the standardized effect size index of functional diversity (SES-FD) of the aboveground traits resulted in a significant effect only for nonlinear components of the model (Table 1). The effect was quite consistent between the two types of sites (‘type’- based on the abundance of the subordinate species *Melica uniflora* or *Carex pilosa*) having similar slope trend, but different intercepts (Figure 1).

For plant height (H) functional diversity, we found a similar linear and nonlinear significant relationship with the increasing coverage of *A. ursinum* (Marginal R^2^ = 16%, Conditional R^2^ = 23%, Table 1), additionally, similar trends on intercepts were consistent between the types of sites (Figure 1a). On contrary, leaf dry matter content (LDMC) resulted in a statistically insignificant linear and nonlinear relationship with *A. ursinum* coverage (Table 1). For leaf area (LA), we had significant linear and nonlinear effects of *A. ursinum* coverage with different values of SES-FD between the two types of sites (different intercepts) (marginal R^2^ = 6%, conditional R^2^ = 53%, Table 1, Figure 1b), although the relationship was consistent between the two types of sites (similar slope variation). Finally, for seed mass (SM) functional diversity, we found a significant linear relationship with *A. ursinum* increasing coverage (Table 1) with a similar effect between two sites (marginal R^2^ = 13%, conditional R^2^ = 19%; Figure 1c).

Regarding the clonal traits, we found a significant nonlinear relationship between *A. ursinum* coverage and the SES-FD of persistent clonal growth organs (PCGO; Table 1, marginal R^2^ = 15%, conditional R^2^ = 47%), though it was quite steady between the two types of sites showing similar slope trend but different intercepts (Figure 1d). Lateral spread and number of clonal offspring (LS and NCO) did not show any significant relationship with *A. ursinum* coverage (Table 1). 

Finally, for leaf dry matter content, plant height, and seed mass (LHS) multiple traits we found significant linear and nonlinear effects of *A. ursinum* coverage (Marginal R^2^ = 18%; Conditional R^2^ = 21%; Table 1) as well as for the overall multiple traits (Marginal R^2^ = 19%; Conditional R^2^ = 34%; Table 1). Both have similarly consistent trends of slopes and intercepts between the types of sites (Figure 1e,f). 

### 2.2. Floristic Composition Comparison

**Figure 2:**
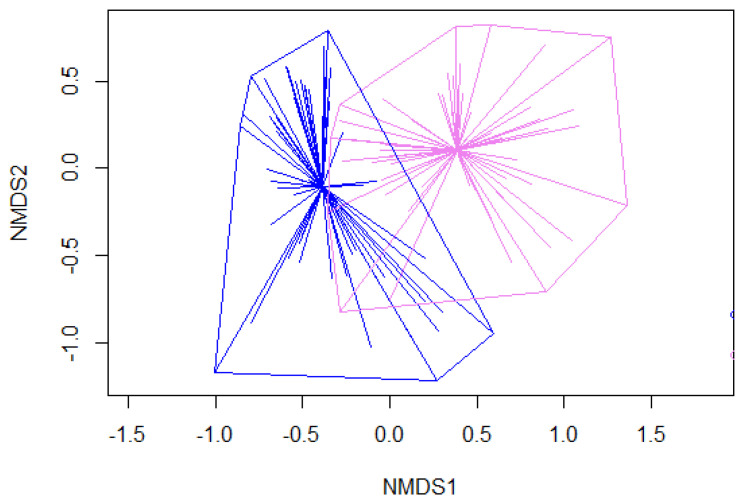
The NMDS graph highlights the floristic dissimilarity between the two types of sites, calculated with Bray-Curtis distance. Blue refers to the presence of the *C. pilosa* type, while pink refers to the presence of the *M. uniflora* type.

The floristic composition comparison was performed to test the dissimilarity or similarity between the two types of sites using the ANOSIM analysis, i.e., to what extent two types of sites differed in their floristic compositions, we found an R-value of 0.4 (*p*-value < 0.001) suggesting that the two types of sites had quite dissimilar floristic compositions (less overlapping; Figure 2).

To assess whether the two types of sites are differently affected in terms of functional trait diversity by *A. ursinum* coverage, we considered aboveground and clonal traits separately and combined. No significant effects were detected between the two types of sites. *A. ursinum* coverage has similar effects on the functional patterns of the coexisting species pool regardless of the changes in species compositions (Figure 1 and Figure 2).

## 3. Discussion

**Figure A1:**
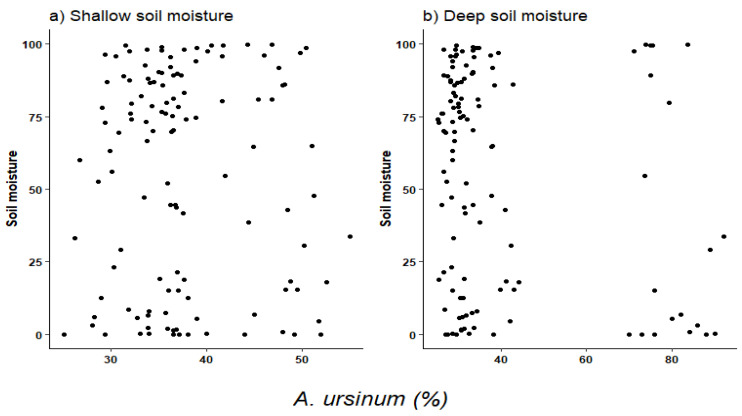
Kendall correlation between *A. ursinum* coverage and soil moisture measured at the depths: (**a**) 7 cm, and (**b**) 20 cm. In both cases tau coefficient values were not significant showing respectively 0.007 (*p*-value = 0.91) and −0.06 (*p*-value = 0.29).

The understory of temperate mature forests is widely considered a stressful environment in terms of resource availability, mainly owing to the limited availability of light [4,9,58]. Accordingly, the habitat filtering processes in such environmental circumstances may result in bearing a specialized or functionally similar flora on an understorey level [5,59]. We adopted carefully selected sampling methods to ensure the least amount of abiotic gradient along the transects on each study site, which was confirmed by analysing the results of soil moisture measurements and ecological indicators values (EIVs) across all the sites (see the methods section and the Table A1, Figure A1 respectively). This allowed us to study the changes in the functional properties related to the interspecific competition alongside the biotic gradient (i.e., increasing *A. ursinum* coverage) [24,31,60]. These results highlight a noticeable sensitivity of understory community assemblages regarding the biotic interactions. 

The increasing *A. ursinum* coverage (25–75% in transition zones; see Methods) indicates an intermediate competition intensity, as the functional diversity reaches its maximum here, while it changes from negative to positive SES-FD values (of H, LA or FD_LHS_ etc.) in the presence of *A. ursinum*. These zones may harbour species that have not appeared anywhere along the whole length of the transects (unpublished work). Further increment in *A. ursinum* coverage leads to the establishment in its monodominance, reducing the interspecific competition to the lowest level, which results in the lowest functional diversity (Figure 1a–c,e). This indicates the weakening of habitat filtering here, which could further escalate into the homogenization of the whole community [48,49,50,51,52,53,54,55].

As is evident from previous studies (launched to investigate functional convergence or divergence; [10,31,37,38,39]), a possible way to coexist with dominant competitor species is either by competing, tolerating or avoiding the interactions with it. So, a dissimilar traits’ portfolio which would facilitate the possible diversification of ecological strategies (niche differences) of subordinate coexisting species, could be achieved by adopting a “competitive response traits“ strategy [6,57], which equates to the tolerance or avoidance of the competitive interactions in the first place [6,7]. In our case, niche differences were important drivers for understorey community assembly at the two different habitats (i.e., types of sites) [6,7,8,9,10,11,12,13,14,15,16,17,18,19,20,21,22,23,24,25,26,27,28,29,30,31,32,33,34,35,36,37,38,39,40,41,42,43,44,45,46,47,48,49,50,51,52,53,54,55,56,57,58,59,60,61,62,63]. Because the outcome of interspecific interactions (i.e., biotic interactions) of a dominant competition species with strong *competitive effects traits* (here, *A. ursinum*; [6,7]) would be competitor exclusion if the coexisting species traits’ portfolios have similar values. If it has functionally dissimilar traits values (in our case - see the redundancy analysis in the Supplementary Section), the resulting functional traits’ variations were more in line with the limiting similarity hypothesis [38] rather than the weaker competitor exclusion [31]. Furthermore, we found major effects of increasing *A. ursinum* coverage on the properties of above ground level.

Contrary to general assumptions, clonal traits were found to have a minor role in species assemblage under intense biotic interactions. Presumably, this could be due to the depth of the remarkable roots of *A. ursinum*, as geophyte bulbs are deeply embedded in the soil [51,64,65], while the foliage is physically interacting with the aboveground functional space of coexisting species. Besides, at the scale of the clonal fragment (physically connected individuals) as suggested by [66,67,68], physiological integration affects the competitive response in the clonal plant as this response is averaged out within the whole clonal fragment, seemingly the responses of the integrator species in the plant community composition are found to be slightly reserved in response to *A. ursinum* coverage. Overall, we can say that different aspects of the ecological niche were similarly involved in species assemblage; the similar trends of each trait of different functional dimensions (such as plant height, leaf trait, seed trait, and clonal traits) and the multiple functional dimensions (like FD_LHS_ and FD_Multiple_) reflected the similar influence of *A. ursinum* coverage on all aspects of SES-FDs (Figure 1).

Lastly, as shown by Figure 2, the dissimilarity in the species composition of two types of sites and, as shown by Figure 1, the similar statistical trends for the two types of sites, it can be seen that the effects of *A. ursinum* on the functional dimension was nevertheless the same. This could be due to fact that the dissimilarity in species compositions (Figure 2) does not harbour enough ‘magnitude’ of trait variability in the trait’s portfolios of sub-ordinate coexisting species to the extent that it can counteract the consistent *competitive effects traits* of the dominant species in the community [6,7] (see also detailed redundancy analysis in the Supplementary Information). As we have not accounted for the intraspecific trait variations (ITV) in this study, variations in the functional traits’ values could be due to the variations in species identity or species cover values [34,69]. This highlights the consistent effects of *A. ursinum* (also due to its deep rooting and sprouting habit) on the plant community functional dimension even if the floristic composition changes.

## 4. Materials and Methods

### 4.1. Study Area

**Figure 3:**
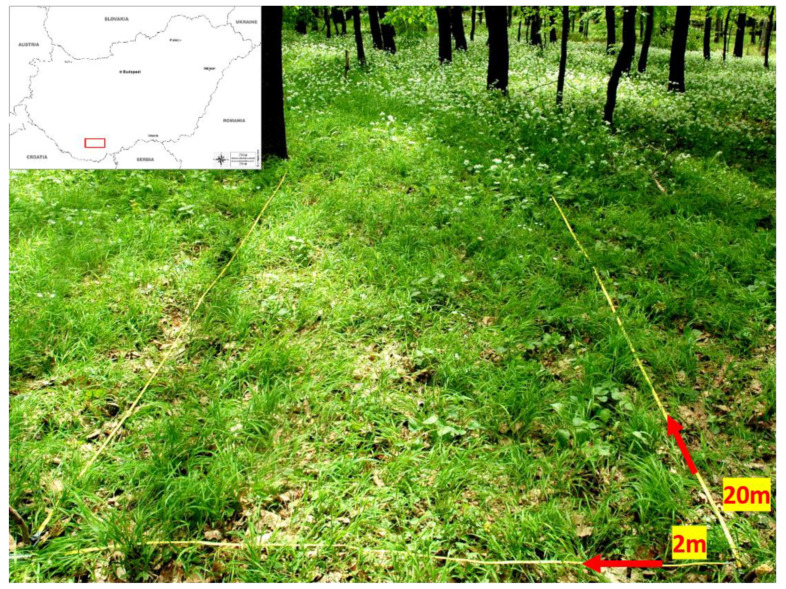
A 2 × 20-m linear transect is laid at one of the study sites. The beginning of the transect has a characteristic abundance of *Melica uniflora*, a transition zone in the middle and a monodominant stand of *Allium ursinum* at the end of the transect. Inset: map of Hungary with study area indicated.

Field studies were conducted in the Mecsek Hills (South Transdanubia Hungary; Figure 3), situated between 46°07′019″ N and 18°14′010″ E at 409 to 475 m a.s.l., where the climate is mostly continental with elements of Mediterranean and Atlantic influence. The area receives mean annual precipitation and temperature of 750 mm and 9.4 °C, respectively. Soil type among sites is mostly brown forest soil with clay illuviation on a Triassic limestone bedrock [54]. Hills are covered with a widespread seminatural, climate-zonal oak-hornbeam forest of *Asperulo taurinae-Carpinetum* Soó *et* Borhidi in Soó 1962 plant association, which mainly occurs in the Mecsek, Villányi and Tolna Hills [70]. In this deciduous mesic forest, the canopy layers are represented by *Quercus dalechampii* Ten., *Carpinus betulus* L., *Fagus sylvatica* L. and *Tilia argentea* DC. etc. The shrub layer is rare and in the herb layer *Allium ursinum*, *Melica uniflora* Retz. and *Carex pilosa* Scop. have characteristic abundances along with several other species in the community.

### 4.2. Vegetation Data

We adopted a fine-scale sampling design to minimise the role that environmental processes may have on the trait patterns i.e., environmental heterogeneity as a process of trait divergence and environmental filtering as a process of trait convergence [71,72]. To examine the possible effects of *A. ursinum* coverage on the functional diversity of coexisting species in its native range; the presence and percentage coverage data of each species per plot were recorded (by visual estimates) during the spring of 2016–2017. A meter quadrat couplet of a size of 2 × 1 m (referred to as a plot here) was repeatedly laid out alongside a 20 m-long linear transect at six sites (in total six transects or 120 plots, see Figure 3). Each linear transect had an absence of *A. ursinum* coverage at the beginning, a transition zone in the middle having increasing *A. ursinum* cover gradient, and finally a monodominant stand of *A. ursinum* at the end of the transect, characteristically resembling a gradient of monodominance across the length of the transect (Figure 3).

Practically, to minimise the environmental heterogeneity (or abiotic gradient) among the sites, the following strategies were adopted for the selection of sites: (i) similar phenological states of *A. ursinum* populations were considered within the only investigated vegetation type of *Asperulo taurinae-Carpinetum* Soó *et* Borhidi in Soó 1962; (ii) young forests or open canopies or shrubby densities; (iii) steep slope of values (>5°); (iv) sharp borders of the *A. ursinum*, *C. pilosa* or *M. uniflora* dominated herb layer; (v) various kinds of animal disturbances and strong human interventions viz. forestry management practices (logging, road construction, shrub cuttings, trampling, harvested areas of *A. ursinum* or other plants etc.); (vi) geomorphological occurrences such as dolines and drainages etc. were all avoided.

To study the *A. ursinum* cover gradient effects on the functional diversity of coexisting species but considering the change in floristic compositions of sites, we selected two ‘types’ of sites based on the abundance of subordinate species at the very beginning of the transect (three sites per type). The first ‘type’ of sites has a characterized abundance of *C. pilosa* while the other ‘type’ of sites has *M. uniflora*.

For the preliminary assessment of variations in ecological conditions (abiotic factors) at the sites, relevant Ellenberg indicator values (henceforth EIV; Ellenberg et al. [73]) were retrieved and adapted for each species according to the Borhidi [74] context of Hungarian flora (Table A1). To consider the shallow and deep roots conditions in the herb layer soil [51], soil moisture data were recorded from each plot at two soil depths (7 and 20 cm) with a time-domain reflectometer (TDR 300 from Spectrum technologies Inc.) during the spring of the same years (2016–2017) after the procurement of vegetation data (Figure A1).

### 4.3. Trait Data

For aboveground traits, the leaf-height-seed (LHS) strategy scheme proposed by Westoby [43], which consists of three independent traits referred to as three different axes of plant functional dimensions, was adopted: LDMC [75,76] as leaf trait represents the resource exploitation strategy [77,78], H is for light competition, and SM for competition among seedlings [44]. Additionally, we included LA, as it captures one functional axis independent of the axis related to LDMC with implications on the regulation of leaf temperature and water-use efficiency during photosynthesis [44]. As for clonal traits we used those that capture largely understudied functional dimensions of space occupancy, resource foraging and sharing such as LS, PCGO and NCO [47,79,80,81].

Species traits values of aboveground and clonal traits were retrieved from LEDA [82,83] and CLO-PLA [46] databases respectively using the *TR8* package [84] in R software (version 3.6.1, R Foundation for Statistical Computing, R Core Team [85]). The *taxize* package was used to update the accepted names of studied flora according to *The Plant List* ([86], Ver 1.1), [87]. The detailed list with definitions of traits is provided in Table A2.

### 4.4. Data Analysis

#### 4.4.1. Assessment of A. ursinum Coverage on the Functional Diversity of Coexisting Species

To assess the effects of *A. ursinum* coverage on the aboveground traits (i.e., LA, H, SM and LDMC) and clonal traits (i.e., LS, PCGO and NCO) of coexisting understory species, we calculated:

- The functional diversity (FD) of every single trait.
- The FD of multiple traits belonging to the LHS scheme (FD_LHS_).
- The FD of all traits (above ground and clonal) pooled together (FD_Multiple_).

We integrated single-trait and multiple-traits analysis since focusing only on the multivariate functional diversity may mask the pattern of community assembly processes [88]. We selected Rao’s quadratic entropy (Q; Rao [89]) as a measure of FD. It measures the expected dissimilarity between two randomly selected individuals of a given assemblage with replacement:

(1) $$Q=\sum_{ij}^{s}d_{ij}p_{i}p_{j}$$

where *S* is the number of species, *d_ij_* is the distance or dissimilarity between the *i*-th and *j*-th species, *p*_i_ and *p*_j_ are the proportions of the abundances of *i*-th or *j*-th species in the sampling unit. The parameter *d_ij_* may vary from 0 (two species bear the same trait values) and 1 (two species bear completely different trait values). Since we aimed to assess the effects of *A. ursinum* on coexistence species and thereby avoiding circularity in the dataset, we excluded *A. ursinum* from the matrix of species composition which was used to compute the FD indices. Then, to analyse if the functional diversity of a given trait was different from random expectation, we calculated the standardized effect size (SES) for each trait separately, by shuffling 999 times the trait values in the ‘species x trait’ matrix and keeping the species composition matrix intact. Then, SES was calculated as follows:

(2) $$SES=\frac{Iobs\;–\;Isim}{\sigma sim}$$

where *I_obs_* is the observed value of the FD, *I_sim_* is the mean of the expected FD, and *σ* is the standard deviation of expected FD. Positive SES values (>0) indicate higher observed values than expected (functional divergence), while negative SES values (<0) indicate lower observed values than expected (functional convergence) and values close to zero means random assembly pattern [41]. This algorithm is suitable for detecting both trait convergence and trait divergence [90].

#### 4.4.2. Modelling the Functional Diversity as a Function of *A. ursinum* Coverage

To assess the effects of *A. ursinum* coverage on the functional diversity of coexistence species we separately fitted mixed models for each of the SES-FD traits in which we included the ‘types’ of sites as a random effect and as a random slope we included the *A. ursinum* coverage. In this way, we were able to account for different values of functional diversity resulting from the difference in the species composition between the types of sites (i.e., different intercept trends) and for different effects of *A. ursinum* due to different species composition (i.e., different slope trend). Finally, we accounted also for nonlinear relationships introducing a quadratic term for the predictor (i.e., *A. ursinum* coverage).

To estimate the explained variance of the model, both conditional and marginal R2 values were calculated. Conditional R2 accounts for the explanatory variance explained by both fixed and random effects, whereas marginal R2 accounts only for fixed effects ([91], Table 1). Model assumptions (normality, homoscedasticity, and independence of the residuals) were graphically evaluated [92].

#### 4.4.3. Assessments of Variations in Floristic Compositions

To quantify and test the variations in species composition between the two types of sites, we performed the analysis of similarity (ANOSIM; with 999 permutations) based on the dissimilarity matrices calculated with Bray–Curtis distance on untransformed coverage data. R values (Correlation coefficient) of ANOSIM may vary from 0 (identifying highly similar groups) to 1 (identifying highly dissimilar groups) [93]. We graphed the floristic dissimilarity computed from ANOSIM using the nonmetric multidimensional scaling (NMDS) (Figure 2). In parallel, a preliminary analysis was performed to test the relationship between ecological features along the length of transects at all the sites ([24], see the Appendix A for details).

All the analyses were done in the R environment [85]. Functional diversity (FD) was computed with the *Rao* function provided in de Bello et al. [94] considering the Jost correction. Mixed models were performed using the *lme* function in the *nlme* package [95]. The conditional and marginal R^2^ were calculated with *r. squared GLMM* function (*MuMIn* package) [96].

## 5. Conclusions

The trait trends of coexisting species for *A. ursinum* coverage are peculiar, with *A. ursinum* monodominance between two types of sites, where the aboveground traits exhibited a varied profile in response to *A. ursinum* cover gradient and clonal traits were found to be more reserved. To emphasise this, we aligned our views to the need as advocated and highlighted by Bittebiere et al.’s review [69] to explicitly include clonal traits in biotic as well as multitrophic level interactions as a promising way to predict species response to the changes in biotic interactions.

As mentioned earlier, after initial coverage fluctuations, *A. ursinum,* along with other shade-tolerant and nutrient loving species was responsible for biotic homogenisation in Germany as it expanded through mesophilic limestone beech forests while substantially altering floristic compositions in both managed and unmanaged stands as reported from five vegetation plots monitored and surveyed over decades [97,98] However, as evident from functional diversity analysis, the niche differences of coexisting species are viable ways to coexist with a competitive and dominant species. Therefore, to preserve biodiversity in the temperate forest herb layer, there must be an ample amount of environmental heterogeneity. As even in tropical forests, landscape heterogeneity was documented to be of great significance in the preservation of understory plant species richness and functional diversity [99]. This significant expansive behaviour of *A. ursinum* should be a good reflection on the understorey species dynamics, while the magnitude of its steady impact on the understorey community assemblies’ processes points out the coherent need to examine understudied biotic interactions in various forest settings.

Furthermore, we recommend studies involving the different types of sites with characteristics abundances of other species, which should shed more light on the behaviour and effects of *A. ursinum* (or alike expansive species) on the functional diversity of various kinds of coexisting species in forest herb layers. While the interplays of abiotic and biotic factors are particularly hard to dissect in field conditions, the best insights can be achieved by conducting similar future investigations both in the field as well as also experimental conditions. 

## Figures and Tables

**Table 1:** Results of mixed model investigating the effects of *A. ursinum* cover gradient on the functional diversity of traits belonging to coexisting species.

Trait Name	Trait Code	Linear Term	Quadratic Term	Marginal R^2^	Conditional R^2^	AIC
Whole-plant trait	H	4.25 ***	2.73 ***	16%	23%	351
						
Leaf traits	LDMC	−0.64 ^n.s.^	0.76 ^n.s.^	2%	22%	213
	LA	1.7 ***	1.14 *	6%	53%	201
						
Seed trait	SM	1.33 ***	0.55 ^n.s.^	13%	19%	98
						
Clonal traits	PCGO	0.29 ^n.s.^	1.83 ***	15%	47%	90
	LS	−0.29 ^n.s.^	0.58 ^n.s.^	1%	37%	107
	NCO	−0.72 ^n.s.^	−0.05 ^n.s.^	2%	69%	25
						
Multiple	LHS	3.19 **	3.49 ***	18%	21%	324
	All traits	4.28 *	3.27 ***	19%	34%	323

The aboveground functional trait dimension is represented with H, plant height; LDMC, leaf dry matter content; LA, leaf area; SM, seed mass, clonal functional trait dimensions with PCGO, persistent clonal growth organ; LS, lateral spread; NCO, number of clonal offspring, aboveground multiple traits with LHS, for leaf dry matter content, plant height, and seed mass and overall multiple traits with all traits that are considered in this study. We reported the best model resulting from a comparison between linear and nonlinear models according to the AIC criterion estimated with the maximum likelihood method (ML). For each mixed model, we reported the coefficient value of fixed effect, marginal, conditional R^2^ values, and AIC values. All parameters were estimated with the restricted likelihood (REML) method. The level of significance for each fixed effect are represented as follows: *** *p* < 0.001; ** *p* < 0.01; * *p* < 0.05; ^n.s.^—not significant.

## Data Availability

A good portion of data that support the findings of this study are available from LEDA, CLO-PLA and Lhotsky et al. [83] but restrictions apply to the availability of these data; hence are not publicly available. Besides, all data generated or analysed during this study are included in this published article. However, with permission of above mentioned third party’s data are available from the corresponding author upon reasonable request. The Supplementary Files are accessible here (https://doi.org/10.5281/zenodo.5588380).

## Supplementary Materials

The following are available online at Zenodo (https://doi.org/10.5281/zenodo.5588380).

## Appendix A

### Ecological Variations Testing Alongside the Length of Transects

To investigate the variations in the ecological factors along the lengths of linear transects with the *A. ursinum* cover gradient; we calculated the community-unweighted mean (CM) of EIV [73]) adapted for the Hungarian flora by Borhidi [74] as follows:

(A1) $$\mathrm{CM}=\sum_{i=1}^{s}p_{i}x_{i}$$

where CM is the community-unweighted mean value of a given EIV, S is the number of species, *p_i_* is the relative abundance of species, *i* (*i* = 1,2, … S), and *x_i_* is the EIV value for species *i*. Since we used species presence data (excluding *A. ursinum*), we have *p_i_* = 1/*N* for all *N* species in the plot. Then, we tested the correlation between each of EIV and *A. ursinum* coverage performing a Pearson correlation test. To correct Type I error, we used the approach suggested by Zelený and Schaffers [100]. We shuffled the EIV across species 999 times and every time we calculated an expected community-unweighted mean to correlate to *A. ursinum* coverage. To assess whether the observed correlation coefficient is significantly different from those expected by chance, we compared the observed correlation coefficient with a distribution of 999 randomly generated expected coefficient values using a two-tailed t-test based on α = 0.025 (Table A1).

Further, we analysed the eventual correlation between *A. ursinum* coverage and soil moisture at the two considered depths by performing a non-parametric Kendall correlation test. (Figure A1) The correlation of community-unweighted mean for EIV with the *A. ursinum* coverage was computed with the function *test_cwm* in the *weimea* package [101]. Kendall correlation between soil moisture and *A. ursinum* coverage was performed with *cor.test* in *stat* package in R [85].

The results of correlation analysis between EIV of sites and *A. ursinum* cover gradient in all the transect did not provide any significant correlation (Table A1). Similarly, we did not find any correlation between *A. ursinum* coverage and soil moisture at the depths of 7 cm (0.007 tau coefficient; *p*-value 0.91) and 20 cm (−0.06 tau coefficient; *p*-value 0.29) (Figure A1).

**Table A1:** Correlation coefficient (*r*) between *A. ursinum* coverage and EIV.

EIV	R	*p*-Value
T	−0.09	0.652
F	0.08	0.698
R	0.16	0.446
N	0.22	0.263
L	−0.06	0.787
C	−0.20	0.341

Values are adjusted to Hungarian flora by Borhidi [73] as temperature figures (T), soil moisture figures (F), soil reaction figures (R), soil nutrients figures (N), light figures (L). Significance deviation of observed coefficient values from a distribution of 999 expected mean values were reported in the *p*-value as follows: ****p* < 0.001; ***p*< 0.01; **p*< 0.05; ^n.s.^ not significant.

**Table A2:** List of plant traits considered in this study with their codes and definitions as present in LEDA database for aboveground traits [82] and CLO-PLA database for clonal traits [46].

Plant Trait	Plant Trait Code	Trait Definition	Relative Coverage of Species with Trait Values across All Datasets
Leaf dry matter content	LDMC	The ratio between oven-dry leaf mass (mg)and its water-saturated fresh mass (g)	86%
Leaf area	LA	The one-sided or projected area of an individual leaf (mm^2^)	87%
Plant height	H	Distance between ground level and the upper photosynthetic vegetative part (cm)	86%
Seed mass	SM	Dry mass of seed without appendices (mg)	87%
Lateral spread	LS	Distance between parental and offspring shoots (cm/year)	94%
Persistence of clonal growth organs	PCGO	Lifespan of the physical connection between mother and daughter shoots (year)	94%
Clonal offspring	NCO	Number of offspring shoots produced per parent shoot per year (n/year)	94%

The relative frequencies of species for which trait data were available and scored at least 80% of the total species coverage considering all the dataset is reported here. This trait threshold is suggested for areas where there is a low turnover of species (i.e., beta diversity; Swenson et al. [102], as was the case in our study (β diversity values of 2.00 expressed as β = γ/mean α considering Jost correction and ‘weighted factor’ as recommended by de Bello et al. [94].
